# The role of advanced imaging in the diagnosis and management of scimitar syndrome in pediatric patients

**DOI:** 10.1016/j.ijcchd.2026.100678

**Published:** 2026-04-16

**Authors:** Thao V.N. Nguyen, Hicran Gul Emral, Inga Voges, S. Yen Ho, Rigby L. Michael, Piers E.J. Daubeney, Edward Nicol, Simon Padley, Cemil Izgi, Raad Mohiaddin, Dudley J. Pennell, Thomas Semple, Sylvia Krupickova

**Affiliations:** a Cardiology Department, Children's Hospital 1, Ho Chi Minh city, Viet Nam; b Department of Pediatric Cardiology, Royal Brompton Hospital, Guy's and St Thomas' NHS Foundation Trust, London, UK; c Department of Congenital Heart Disease and Pediatric Cardiology, University Hospital Schleswig-Holstein, Kiel, Germany; d DZHK (German Center for Cardiovascular Research), partner site Hamburg/Kiel/Lübeck, Germany; e National Heart & Lung Institute, Imperial College London, UK; f Radiology Department, Royal Brompton Hospital, Guy's and St Thomas' NHS Foundation Trust, London, UK; g King's College London, UK; h Cardiovascular Magnetic Resonance Unit, Royal Brompton Hospital, London, UK

**Keywords:** Scimitar syndrome, CMR, Cardiac CT

## Abstract

This imaging review highlights the spectrum of Scimitar Syndrome (SS) as seen on cardiac computed tomography (CT) and cardiovascular magnetic resonance (CMR), illustrating its variants and linking imaging findings with clinical presentation and management strategies.

SS is a rare congenital cardiopulmonary anomaly characterized by partial or total right anomalous pulmonary venous drainage to the hepatic vein or inferior vena cava (IVC), hypoplastic right lung and pulmonary artery, abdominal aortopulmonary collateral(s) to the right lower lobe and other cardiac and bronchial malformations. Management options range from surgical lobe resection, rerouting of the anomalous pulmonary vein to the left atrium, catheter-based embolization of aorto-pulmonary collaterals, and intracardiac shunt device closure, to conservative observation. While surgical outcomes are generally favorable, postoperative complications can be substantial, and the indications for surgery—especially in asymptomatic patients—remain unclear and should be individualized. A multimodal imaging approach is essential for accurate diagnosis, hemodynamic assessment, and follow-up, enabling tailored treatment planning. This manuscript presents role of cardiac CT and CMR using representative imaging examples, highlighting the diagnostic value and discussing implications of the findings for clinical management and longitudinal follow-up.

## Highlights

• Scimitar syndrome shows a broad spectrum of anatomical variants.
• CT and CMR provide complementary anatomical and functional data.
• CT enables rapid, high-resolution anatomical assessment in infants.
• CMR enables longitudinal follow-up and hemodynamic assessment.

## 1 Introduction

Scimitar Syndrome (SS) is a rare congenital anomaly typically characterized by the anomalous pulmonary venous drainage of all or part of the right lung to the inferior vena cava (IVC) or hepatic vein, often associated with right lung hypoplasia, and systemic arterial supply to the right lung segments usually arising from the abdominal aorta close to the celiac axis. More than one collateral is occasionally seen. Secundum atrial septal defect (ASD) is sometimes found and a right sided heart often described as dextroposition is the usual finding [1]. Presentation ranges from incidental diagnosis in asymptomatic children or adults to severe congestive heart failure or pulmonary artery hypertension (PAH) in infancy. Diagnosis can be suspected via a chest X-ray, but confirmation is often achieved through more advanced imaging such as echocardiography, computed tomography (CT), or cardiac magnetic resonance (CMR). While many patients are asymptomatic and can be managed conservatively, those with complications such as congestive heart failure, PAH or a high pulmonary-to-systemic blood flow ratio require surgical rerouting of the anomalous pulmonary vein(s). The indications for surgical repair of SS remains unclear, and lacks evidence, especially for asymptomatic patients. Surgical outcomes are generally favorable, but postoperative complications remain significant [2,3]. The 2025 American College of Cardiology/American Heart Association Joint Committee on Clinical Practice Guidelines advocate for a personalized approach [4], with multimodality imaging playing a crucial role in determining the timing of surgery and customizing treatment plans. This review highlights the spectrum of SS as seen on CT and CMR, illustrating its variants and linking imaging findings with clinical presentation and management strategies.

## 2 Clinical presentation

SS can be diagnosed in fetal life, but the usual clinical spectrum is from severely ill infants to asymptomatic children or adults, with presentations having been artificially classified into three categories: an infantile form with symptoms, heart failure and PAH, an “older” adult form distinguished by being asymptomatic in infancy, and a form with associated congenital cardiac anomalies [5].

In the infantile form, respiratory and/or cardiac failure is often due to PAH associated with cardiac and/or right lung anomalies. PAH is more common in patients with additional congenital heart defects or an anomalous large systemic arterial supply, and less frequently due to pulmonary vein stenosis. Interventional treatments such as embolization of systemic arterial collaterals and ASD closure can reduce shunt volume, alleviate PAH, and prevent the progression of heart failure in infants. However, the prognosis in infantile SS remains guarded, with or without interventional treatment or surgery [[1], [2], [3]].

The childhood/adult forms are typically asymptomatic in infancy, have better long-term outcomes. Studies show that symptomatic children and adults, presenting with recurrent respiratory infections or exercise intolerance, often improve significantly following surgical correction. Surgery is indicated for asymptomatic patients with a pulmonary-to-systemic blood flow ratio (Qp:Qs) > 1.5 to prevent complications such as PAH [4]. However, due to the high incidence of subsequent scimitar vein stenosis, IVC stenosis, and thrombosis, the indications for surgery in asymptomatic patients remain controversial, with differences in management between centers [[2], [3], [4]].

At any age, recurrent respiratory infections—frequently involving the right lower lobe—are common and are thought to result from abnormal bronchial branching within the hypoplastic right lung, anomalous pulmonary vasculature, and secondary PAH [6,7]. The frequency and severity of infections correlate with the degree of pulmonary hypoplasia, and rarely lobectomy or pneumonectomy have been performed to manage severe bronchiectasis or chronic pulmonary infections in the sequestrated lobe [[8], [9], [10]]. Hemoptysis can also occur due to PAH, anomalous aorto-pulmonary collaterals or bronchiectasis [[11], [12], [13]]. In many cases, SS is discovered incidentally, such as through the detection of a heart murmur or abnormalities on chest X-rays.

## 3 Imaging modalities for evaluation of scimitar syndrome

**Fig. 1:**
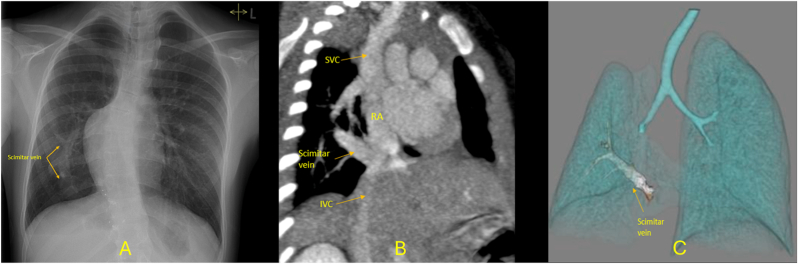
(A) Chest X-ray from a 15-year-old girl shows a scimitar vein parallel to the right heart border in the shape of Turkish sword (Scimitar). (B, C) Images of a 6-month-old girl with SS with abnormal bronchial branching with a hypoplastic single lobed right lung: (B) Oblique CT reconstruction shows the scimitar vein from the right lung draining into the IVC at the inferior cavo-atrial junction; (C) 3D volume and surface rendered image shows abnormal bronchial branching with a hypoplastic single lobed right lung and the scimitar vein. SS: Scimitar Syndrome, CT: Computed Tomography, IVC: Inferior Vena Cava, 3D: Three-Dimensional.

The triad of respiratory distress, right lung hypoplasia, and dextroposition of the heart secondary to reduced right lung volume should prompt clinicians to consider SS [5]. Chest X-ray with a “scimitar sign” and transthoracic echocardiography (TTE) are often the first-line imaging modalities used in the diagnosis of SS, especially in detecting associated lesions such as ASD and ventricular septal defect (VSD) (Fig. 1A). However, these methods are limited in visualizing the pulmonary veins and associated vascular anomalies. In such cases, cross-sectional techniques like CT or CMR are often required for more detailed anatomical evaluation. This article focuses on the role of CT and CMR in the diagnosis and management of SS, emphasizing their contributions to comprehensive assessment.

ECG-gated CT is particularly valuable for anatomical assessment in SS, providing high-resolution images of the heart, great vessels, coronary arteries as well as the airways and lung parenchyma. It excels in visualizing pulmonary venous drainage, including the presence of a scimitar vein and anomalous venous connections, as well as identifying structural abnormalities such as hypoplastic lungs and abnormal bronchial branching (Fig. 1B, 1.C). Additionally, CT is highly effective for evaluating associated anomalies, including coronary artery anomalies, and provides essential information on aortopulmonary collaterals (APCs) that are commonly seen in SS [14]. Additional associations of SS that are well depicted by CT include diaphragmatic hernia, lung sequestration, horseshoe lungs and tracheobronchial stenosis [[15], [16], [17], [18]]. CT's superior temporal and spatial resolution is especially beneficial in small patients with respiratory symptoms or high heart rates, often enabling comprehensive evaluation of the cardiovascular and pulmonary systems of infants and small children without the need for general anesthesia or sedation. In terms of hemodynamic evaluation, assessment of biventricular volumes and function, as well as calculation of Qp:Qs using continuous acquisition throughout the cardiac cycle and the test bolus-derived Qp:Qs technique has been previously described [19,20] but not widely used in clinical practise.

CMR is the non-invasive gold standard tool in evaluating hemodynamics in SS, especially for quantifying left-to-right shunt by assessing the Qp:Qs ratio caused by anomalous pulmonary venous connections and intracardiac shunt, and “split flow” through each branch pulmonary artery. CMR plays a critical role in evaluating ventricular volumes and function, providing detailed insights into both right and left ventricular performance and volume overload. The 3D contrast-enhanced MR angiography and 3D balanced Steady-State Free Precession (bSSFP) whole heart images are particularly useful in SS for assessing complex anatomical features such as scimitar vein drainage and vascular anomalies with high spatial resolution (around 1 mm). Additionally, CMR with stress perfusion and late gadolinium enhancement (LGE) sequences is beneficial in distinguishing between ischemic areas and infarcted tissue associated with coronary artery anomalies, which may occasionally coexist with SS. CMR is also highly valuable for follow-up evaluation, allowing for reassessment of complications such as scimitar vein stenosis, IVC stenosis, and redistribution of pulmonary arterial flow [[21], [22], [23], [24]]. This makes it an excellent tool for monitoring surgical outcomes and optimizing the timing for future interventions or surgery.

## 4 Role of imaging in preoperative and postoperative assessment

### 4.1 Preoperative planning

Both CT and CMR allow accurate assessment of the anatomy of the scimitar vein. These imaging modalities help identify the specific course of the anomalous pulmonary venous drainage, evaluate the number and position of the scimitar vein termination, determine the presence of scimitar vein stenosis, presence of APCs and intracardiac shunts all of which are crucial for guiding interventional and surgical planning (Table 1).

**Table 1:** Key anatomical features of scimitar syndrome variants relevant to treatment planning and the role of advanced imaging. PV: pulmonary vein; RV: right ventricle; ASD: atrial septal defect; CT: computed tomography; CMR: cardiovascular magnetic resonance.

Anatomical Features	Clinical Implications and Management	Role of Advanced Imaging Modalities
**Scimitar vein**		**CT:** Detailed delineation of pulmonary venous anatomy, especially in infants and young children **CMR:** Assessment of PV flow (visualization and quantitative analysis) Differential pulmonary blood flow (RPA:LPA) Shunt quantification (Qp:Qs) RV volume and function
Drainage site and length	PV rerouting surgery	
Scimitar vein stenosis	High association with post-surgical PV stenosis → may require scimitar vein stenting or PV augmentation techniques	
Dual drainage (e.g., with meandering vein)	Consider scimitar vein occlusion after balloon occlusion testing confirms adequate alternative drainage (e.g., via meandering vein)	
**Large aortopulmonary collaterals**	Transcatheter embolization of collaterals	**CT:** Detailed anatomical mapping of collateral vessels **CMR (pre- and post-procedure):** Hemodynamic assessment (Qp:Qs) RV volume and function to evaluate treatment response
**Additional intracardiac shunt (e.g., ASD)**	ASD closure alone with conservative management of the scimitar vein in selected cases or surgical ASD closure and rerouting of pulmonary veins.	**CMR:** Quantitative flow assessment to determine hemodynamic significance of ASD and scimitar vein Follow-up evaluation of Qp:Qs after ASD closure
**Right lung hypoplasia and tracheobronchial abnormalities**	Surgical resection (lobectomy or pneumonectomy) in selected cases	**CT:** Detailed evaluation of lung parenchyma and airway anatomy

**Fig. 2:**
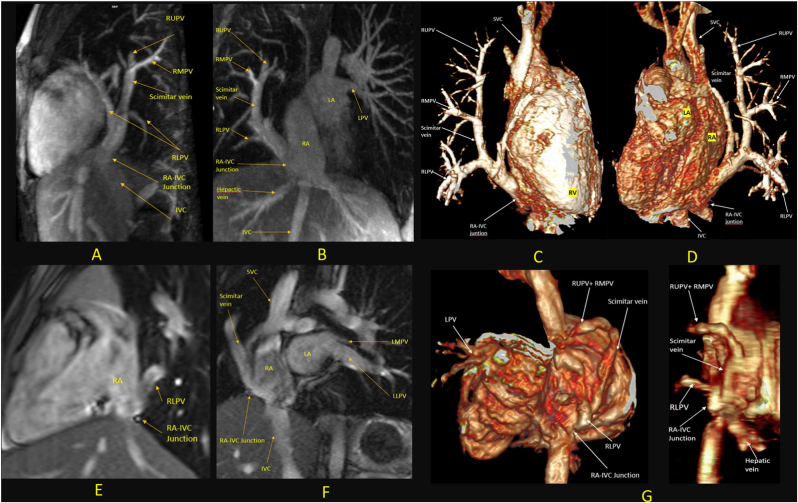
Two types of scimitar veins. (A, B, C, D) Single scimitar vein. CMR images from a 12-year-old asymptomatic girl with SS. 3D contrast-enhanced MR angiography and reconstructed images show all right pulmonary veins joining together and draining through a single scimitar vein into the inferior cavo-atrial junction without any stenosis. (E, F, G) Two scimitar veins. CMR image from an 8-year-old girl with asymptomatic SS. TRUFI Coronal, Sagittal sequences and 3D Reconstructed images from 3D SSFP images show the RUPV and RMPV joined together and draining through the scimitar vein into the inferior cavo-atrial junction while the RLPV drained separately into the inferior cavo-atrial junction, just below the exit point of the scimitar vein without any stenosis. CMR: Cardiac Magnetic Resonance, TRUFI: True Fast Imaging with Steady-State Precession, RUPV: Right Upper Pulmonary Vein, RMPV: Right Middle Pulmonary Vein, RLPV: Right Lower Pulmonary Vein, RV: Right Ventricle.

Two types of scimitar veins have been recognized: the classic simple scimitar vein, which runs from the middle of the right lung to the cardiophrenic angle and a variant with two scimitar veins (Fig. 2) [[25], [26], [27]]. The scimitar vein(s) termination can be IVC, part of azygos system, hepatic vein or other systemic veins [28]. In cases of two scimitar veins, the inferior anomalous pulmonary vein may drain into IVC while the superior one drains into the superior vena cava [27].

**Fig. 3:**
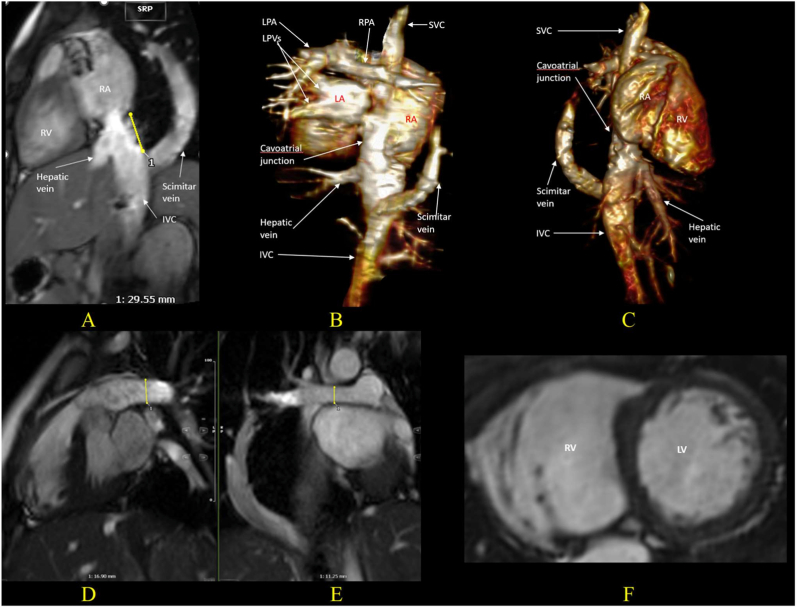
CMR images from an asymtomatic 15-year-old girl with SS and scimitar vein termination into the IVC below the hepatic vein level, previously treated with catheter-based embolization of APCs for follow-up evaluation:(A, B, C) TRUFI sagittal image and 3D reconstructed images from 3D bSSFP whole heart data reveal total anomalous right pulmonary venous return with a scimitar vein draining into the IVC below the hepatic veins, 30 mm inferior to the inferior cavo-atrial junction. (D, E) bSSFP cine images in end-systolic phase show discrepancy in diameter between RPA (diameter 11 mm) and LPA (diameter 17 mm) with a 38%: 62% RPA: LPA flow split. (F) bSSFP short axis cine images in end-diastolic phase show a dilated RV (with RVEDV of 112 ml/m^2^, RVESV of 47 ml/m^2^, and preserved RVEF of 58%). 2D phase contrast sequences through the aorta at STJ junction and MPA (not shown here) show modest left-to-right shunt (Qp:Qs = 1.5:1). Given that the scimitar vein termination below the hepatic vein level poses a significant technical challenge for surgical repair due to the large distance between the left atrium and the scimitar vein, borderline Qp:Qs, borderline RVEDV and the patient's stable clinical status, a conservative approach was taken, opting for close monitoring without surgical intervention. CMR: Cardiac Magnetic Resonance, TRUFI: True Fast Imaging with Steady-State Precession, bSSFP: balanced Steady-State Free Precession, IVC: Inferior Vena Cava, RUPV: Right Upper Pulmonary Vein, RMPV: Right Middle Pulmonary Vein, RLPV: Right Lower Pulmonary Vein, RV: Right Ventricle, RVEDV: Right Ventricular End-Diastolic Volume, RVESV: Right Ventricular End-Systolic Volume, RVEF: Right Ventricular Ejection Fraction, Qp:Qs: Pulmonary-to-Systemic Blood Flow Ratio.

In cases where the termination of the scimitar vein is located far from the inferior cavo-atrial junction, the significant distance between the scimitar vein and left atrium (LA) poses a significant challenge for surgical repair (Fig. 3) [29]. Consequently, direct anastomosis of the scimitar vein to LA without any stenosis of pulmonary or systemic venous return may be technically challenging.

Preoperative detection of scimitar vein stenosis is essential, as it is associated with early clinical presentation, a higher prevalence of pulmonary hypertension, and worse outcomes [29]. Various surgical techniques have been employed to relieve pulmonary venous stenosis and reduce the risk of postoperative obstruction, including fibrous crest resection and oblique incision of the scimitar vein [29]. However, the incidence of postoperative pulmonary vein stenosis in this population remains high [29,30].

**Fig. 4:**
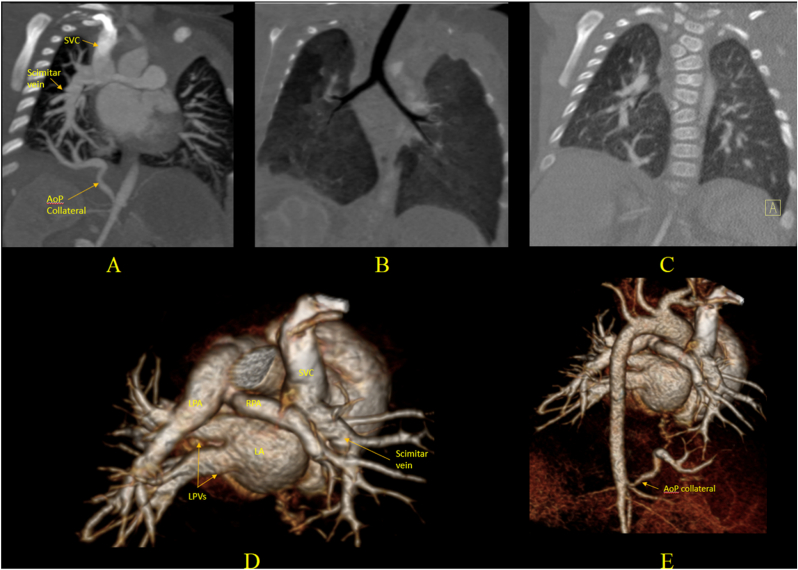
ECG-gated CT images of 3-month-old boy with post-natal diagnosis of partial anomalous pulmonary venous drainage. (A) Coronal image shows the heart and mediastinum are shifted to the right due to mild hypoplasia of right lung. The right pulmonary venous drainage is entirely to the superior vena cava by an unusual vessel that looks like an “inverted scimitar vein”. An APC arises from the abdominal aorta, close to the origins of the celiac axis and superior mesenteric artery, stenosed at its origin supplying the base of the right lung. (B) The right lung comprised two lobes only with abnormal branching of the right bronchus. (C) Coronal image shows vertebral segmentation anomalies within the spine. (D, E) 3D volume rendered images show again the inverted scimitar vein to SVC, the aortopulmonary collateral, the right pulmonary artery slightly smaller than the left, with diameters 6.5 mm and 8.7 mm respectively. This patient had a catheter intervention for collateral embolization with two 8 mm Amplatzer Vascular Plug IV and five 5 × 5mm coils at 2-years-old and Warden procedure to re-route the scimitar vein to the LA at 3 years-old with good outcome. ECG: Electrocardiogram, CT: Computed Tomography, SVC: Superior Vena Cava, APC: Aortopulmonary Collateral, IVC: Inferior Vena Cava, LA: Left Atrium.

Another variant which has inverted scimitar vein draining to superior vena cava also shares the same characteristics of “scimitar sign” (Fig. 4). Besides, left-sided SS has been reported with the left scimitar vein collecting left pulmonary venous flow, joining the azygos vein, pericardiophrenic vein, left hepatic vein close to the IVC or directly to the IVC, with or without associated left lung parenchymal or bronchial abnormalities [[31], [32], [33], [34]].

**Fig. 5:**
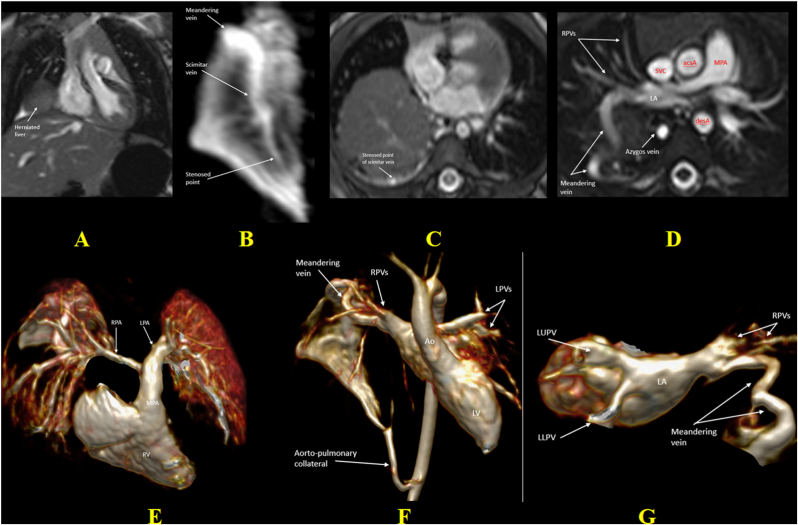
CMR images from an 11-month-old girl with an unusual variant of SS draining into the azygos vein with severe stenosis, patent meandering vein to LA, large aortopulmonary collateral and diaphragmatic hernia, who presented with tachypnoea and feeding difficulties. (A) TRUFI coronal image shows meso-position and levoapex heart, with hypoplastic right lung and herniated liver. (B) CE MR angiogram shows the scimitar vein with a stenosis, and (C) trans-axial bSSFP cine image shows the scimitar severe stenosis (1.5 mm) at level of herniated liver. (D) Trans-axial SSFP cine image at proximal ascending aorta level shows that the right pulmonary venous flow drains back to a patent tortuous meandering vein with some preserved small RPVs normally draining to the LA and a prominent azygos vein. (E, F, G) Reconstruction images from CE 3D angiogram show the hypoplastic RPA (3 mm), larger LPA (7 mm) with a 25%:75% RPA: LPA flow split, a large APC from the abdominal aorta and a tortuous meandering vein draining to the LA. The Qp:Qs of 1.4:1 indicates a mild left-to-right shunt. Given the natural stenosis of the scimitar vein, the effective alternative drainage via the meandering vein, and only mildly increased pulmonary flow, the decision was made to proceed only with catheter-based embolization of the APC to reduce pulmonary over-circulation. The clinical outcome was favorable, with improved feeding, respiratory status, appropriately subsequent growth, and development. CMR: Cardiac Magnetic Resonance, SS: Scimitar Syndrome, LA: Left Atrium, RPA: Right Pulmonary Artery, LPA: Left Pulmonary Artery, RPV: Right Pulmonary Vein, Qp:Qs: Pulmonary-to-Systemic Blood Flow Ratio, bSSFP: Balanced Steady-State Free Precession, TRUFI: True Fast Imaging with Steady-State Precession, CE: Contrast-Enhanced, IVC: Inferior Vena Cava, APC: aorto-pulmonary collateral.

In patients with dual drainage via the scimitar vein and a patent “meandering vein”—an anomalous right pulmonary vein that follows a tortuous route through the lung before draining normally into the LA—intervention using embolization of the scimitar vein and APCs, along with ASD closure if present, may effectively divert right pulmonary venous flow back to the LA and reduce pulmonary blood flow without the need for surgery [35,36]. Balloon occlusion test is required to confirm the adequate pulmonary venous drainage via the meandering vein [37]. Additionally, in cases with natural stenosis of the scimitar vein and a patent meandering vein, the management can often be simplified by APCs occlusion alone, avoiding the need for more invasive surgical intervention (Fig. 5)**.**

**Fig. 6:**
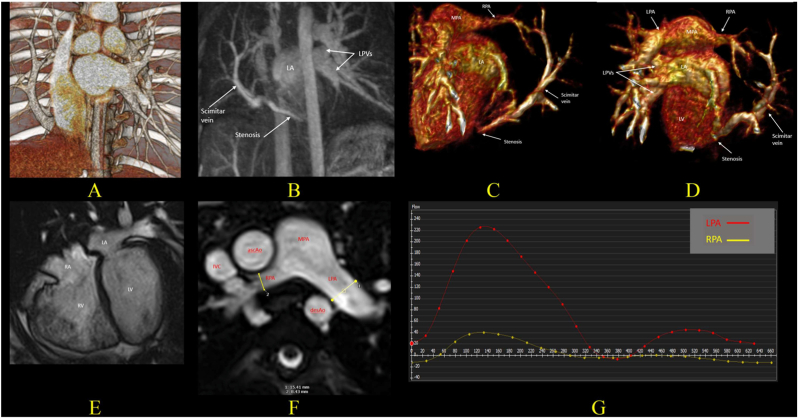
Images from an 8-year-old girl with SS, status post-operative rerouting of the scimitar vein to the left atrium, (A) Preoperative 3D volume rendered CT image reveals all right pulmonary venous drainage to the IVC via a large scimitar vein near the inferior cavo-atrial junction. Due to progressive symptoms and evidence of significant left-to-right shunting despite aortopulmonary collateral embolization, surgical rerouting of the scimitar vein into the left atrium was performed with IVC augmentation using bovine pericardial patch. Postoperatively, the patient developed recurrent pericardial effusion and severe stenosis at the anastomosis, near occlusion, which was resistant to balloon dilation. CMR at 3 years post-surgery was performed to reassess anatomy and hemodynamic. (B, C, D) 3D CE-MRA and reconstructed images show all pulmonary veins joining the scimitar vein, then stenosed at the augmentation patch level before reaching the LA. (E) bSSFP 4-chamber view cine image in the end-diastolic phase shows a dilated RV. (F) bSSFP cine image shows a moderately hypoplastic RPA compared to the LPA (9.4 mm and 15.4 mm, respectively). (G) Flow figure from 2D Phase-contrast sequences through the RPA and LPA shows significant pulmonary flow redistribution. The minority of pulmonary blood flow goes through RPA with diastolic reverse-flow (Yellow line) and majority of pulmonary flow goes to LPA (Red line). RPA: LPA split net flow of 6%:94%. Qp:Qs ratio was 1:1. Conservative management was chosen due to high surgical risk for further intervention. SS: Scimitar Syndrome, IVC: Inferior Vena Cava, LA: Left Atrium, CMR: Cardiac Magnetic Resonance, TRUFI: True Fast Imaging with Steady-State Precession, bSSFP: balanced Steady-State Free Precession, RPA: Right Pulmonary Artery, LPA: Left Pulmonary Artery, Qp:Qs: Pulmonary-to-Systemic Blood Flow Ratio.

CMR, with its accurate quantification of left-to-right shunting and Qp:Qs ratio, is particularly helpful in asymptomatic patients. A Qp:Qs ratio >1.5 is generally considered indicative of hemodynamic significance and alongside dilated right heart chambers may support consideration for intervention in the appropriate clinical context [4]. Split net RPA and LPA blood flow is also a key parameter in the evaluation of SS. Commonly, relative blood flow of <40% to the left pulmonary artery and <50% to the right pulmonary artery are regarded as abnormal [38]. The redistribution of split net RPA and LPA blood flow in scimitar syndrome refers to an imbalance in pulmonary perfusion between the two lungs, characterized by reduced blood flow to the right lung and compensatory increased flow to the left lung, even when the RPA and LPA do not show a significant size discrepancy. This auto-regulation mechanism helps mitigate overflow in the pulmonary system, balancing blood flow distribution. Given the unclear indications for surgery in asymptomatic patients, repeated follow-up CMR plays a crucial role in reassessing hemodynamic significance and right ventricular function, providing essential information for determining the optimal timing for surgery (Fig. 3, Fig. 6).

### 4.2 Postoperative follow-up

In cases with successful surgery or interventional treatment, TTE may suffice for follow-up, provided there is good flow through the scimitar vein and no apparent signs of PAH or RV dysfunction. However, in complicated cases following surgery, follow up with advanced imaging is required.

Postoperative scimitar vein obstruction remains the most significant complication. Obstruction typically involves the length of the intra-atrial baffle, often related to baffle tension or thrombosis, rather than the scimitar vein–atrial junction [29,30]. Transcatheter balloon dilation or stent implantation at the site of stenosis has emerged as an effective therapeutic strategy, particularly given the substantial risks associated with reoperation [39]. However, scimitar vein stent thrombosis must be considered in patients who develop recurrent hemoptysis or PH after stent placement [40]. In these urgent scenarios, cardiac CT is an excellent modality for rapid diagnosis and precise delineation of the baffle tunnel anatomy to guide interventional planning. The presence and position of interatrial communication in the atrial septum also can be described by 3D reconstruction and modeling, helping to plan the intervention approach and the atrial septum puncture [41]. CT is also reserved for ongoing questions regarding particularly neonates or young patients, or in cases with small, tight venous stenosis.

CMR is an effective modality for hemodynamic reassessment after surgery or intervention, enabling evaluation of right ventricular functional recovery, reduction in Qp:Qs, and redistribution of pulmonary blood flow, thereby providing comprehensive information to guide ongoing management (Fig. 6). Patients who undergo transcatheter intervention, including collateral embolization or ASD device closure without scimitar vein correction, demonstrate a reduction in Qp:Qs to less than 1.5 may be managed conservatively with clinical and imaging follow-up, without the need for surgical repair. Although CMR's spatial resolution is generally inferior to that of CT, ongoing technical advances have substantially improved image quality. In clinically stable patients, CMR can also detect postoperative complications, including scimitar vein stenosis, by using 3D contrast angiography. Redistribution of pulmonary blood flow between RPA and LPA, which may occur in cases of unilateral pulmonary vein stenosis, can be assessed by quantifying flow within the branch pulmonary arteries [21].

**Fig. 7:**
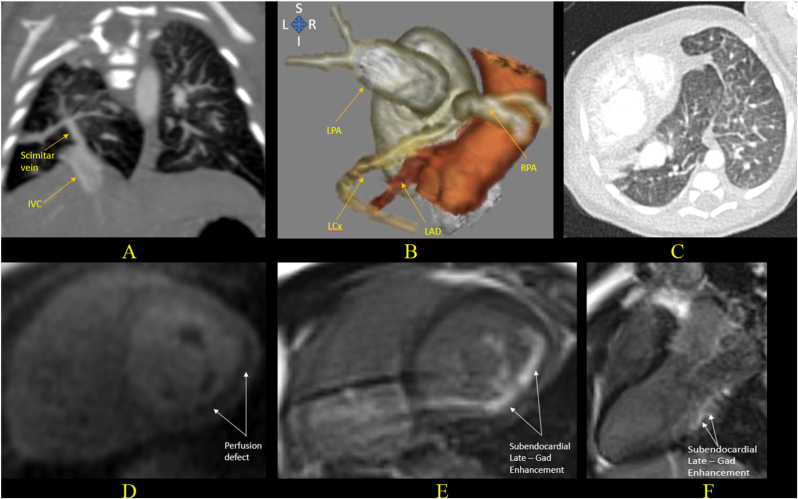
Images of a 6-day-old girl with SS. (A) CT image in the coronal plane shows anomalous drainage of the right lung via a scimitar vein into the hepatic IVC. (B) 3D volume rendered image shows a hypoplastic RPA with measurement 3 mm, compared to the LPA that measured 6.7 mm. The LCx coronary artery arises from the hypoplastic RPA while the LAD arises normally from aorta. (C) CT image in axial plane shows dextroposition of the heart and horseshoe segment of right lung passing between the aorta and heart, appearing confluent with the left lung. This patient had catheter embolization of an aortopulmonary collateral and surgical reimplantation of the LCx coronary artery in infancy. (D, E, F) CMR stress perfusion and late gadolinium enhancement (LGE) images after the surgery at the age of 10 years show a persistent perfusion defect and subendocardial LGE in the infero-lateral wall, basal and mid portion of LV, consistent with the infarction of the LCx territory. SS: Scimitar Syndrome, CT: Computed Tomography, IVC: Inferior Vena Cava, RPA: Right Pulmonary Artery, LPA: Left Pulmonary Artery, LCx: Left Circumflex Artery, LAD: Left Anterior Descending Artery, CMR: Cardiac Magnetic Resonance, LGE: Late Gadolinium Enhancement, LV: Left Ventricle.

**Fig. 8:**
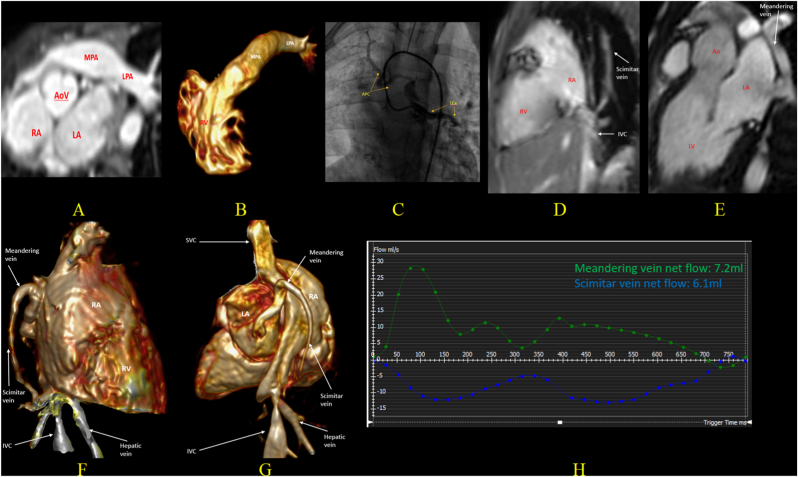
Images from an asymptomatic 14-year-old girl with a known variant of SS with absent right pulmonary artery, APCs from the descending aorta and from the left circumflex artery (LCx) to right lung, who had previously undergone two catheter-based coil embolizations of APCs from the abdominal aorta during infancy, leaving the coronary-pulmonary collateral. A follow-up exercise stress test at age 10 revealed 2.5 mm ST-segment depression in the inferior leads at peak exercise, which resolved within 3.5 min of recovery. Follow-up CMR for evaluating coronary-pulmonary collateral and hemodynamic assessment: (A, B) CMR 3D bSSFP whole heart image and reconstructed image show absent RPA. 2D PC images (not shown here) confirm the MPA flow is equal to the LPA flow. (C) Transcatheter angiogram shows collateral from the LCx supplying the right lung and mildly dilated LCx. (D) TRUFI sagittal and coronal images show the scimitar vein receiving right-sided pulmonary venous flow, draining to IVC, terminating at inferior cavo-atrial junction with severe stenosis. (E) bSSFP LV 3 chamber image shows additional meandering vein connecting scimitar vein to LA. (H, I) Reconstructed images from 3D CE-MRA show again the scimitar vein continuing to meandering vein to the LA, and a stenosis at the scimitar termination point. (K) Flow figure from 2D Phase-contrast images via the scimitar vein and meandering vein show most of the scimitar vein flow drains back into the LA via the meandering vein, rather than returning to the IVC. There was no significant left-to-right shunt with Qp:Qs = 1:1. Importantly, CMR demonstrated hypokinesia of the LV infero-septal wall (mid to apical segments), along with a mild-to-moderate increase in LVEDVi (110 ml/m^2^) and low-normal LVEF (55%), raising concern for a possible coronary steal phenomenon due to the LCA-to-lung collateral. The APCs' flow burden (including the coronary-pulmonary collateral) is calculated to be around 15%. LGE was not performed. SS: Scimitar Syndrome, RPA: Right Pulmonary Artery, APCs: Aortopulmonary Collaterals, LCx: Left Circumflex Artery, IVC: Inferior Vena Cava, LA: Left Atrium, CMR: Cardiac Magnetic Resonance, TRUFI: True Fast Imaging with Steady-State Precession, bSSFP: Balanced Steady-State Free Precession, MPA: Main Pulmonary Artery, LPA: Left Pulmonary Artery, Qp:Qs: Pulmonary-to-Systemic Blood Flow Ratio, LV: Left Ventricle, RV: Right Ventricle, LVEDVi: Left Ventricle End Diastolic Volume Index, LVEF: Left Ventricular Ejection Fraction.

In cases with collaterals from the coronary arteries or coronary origin anomalies such as anomalous left circumflex coronary origin from the pulmonary artery, CT is the optimal non-invasive modality for demonstrating the anatomy pre-operatively and in assessing for suspected post-reimplantation stenosis. CMR with stress perfusion can also help evaluate for ischemic areas following the reimplantation of an abnormally originating coronary artery (Fig. 7, Fig. 8).

## 5 Protocols

If expertise and equipment is available, CT in scimitar syndrome benefits from ECG gating to ensure movement artefact imaging, particularly if the coronary origins are to be delineated. There is also a need for contrast enhancement of both the right and left heart structures alongside the systemic venous (IVC) and pulmonary venous systems. The Royal Brompton's variation of the Bastion wheel contrast protocol achieves this with ease. The Bastion protocol is a biphasic contrast technique employs weight-based contrast dosing (commonly 2 mL/kg) and a split-bolus injection strategy, typically administering one-third followed by two-thirds of the total contrast volume. This approach enables simultaneous arterial and venous enhancement within a single acquisition, thereby reducing radiation exposure and overall scan time. Image acquisition is initiated during injection of the second bolus, capturing arterial enhancement from the first bolus as it reaches the vascular system and venous enhancement from the second bolus.

CMR guidelines and protocols for congenital heart disease have been published by the Society for Cardiovascular Magnetic Resonance [42] and European Association of Cardiovascular Imaging [43]. Our CMR protocol in patients with SS in Royal Brompton Hospital with standard and advanced sequences is displayed in Table 2.

**Table 2:** CMR protocol for patients with Scimitar Syndrome. **Highlighted sequences** for advanced protocols. HASTE: Half-Fourier Acquisition Single-shot Turbo Spin Echo, TrueFISP: True Fast Imaging with Steady-State Precession, bSSFP: Balanced Steady-State Free Precession, LVOT: Left Ventricular Outflow Tract, AO Valve: Aortic Valve, RV: Right Ventricle, RPA: Right Pulmonary Artery, LPA: Left Pulmonary Artery, PC – Phase Contrast, STJ: Sino-Tubular Junction, MPA: Main Pulmonary Artery, CE-MRA: Contrast-Enhanced Magnetic Resonance Angiography, LA: Left Atrium, 3D: 3-Dimensional, LGE: Late Gadolinium Enhancement, APCs: aorto-pulmonary collaterals, PVs: Pulmonary veins, 4D: 4-Dimensional.

Protocol	Sequences	Comments
Scout and localizer		
**Anatomy**	• HASTE Transaxial • True FISP coronal, sagittal, transaxial with no distance gap	Cardiac and extracardiac structures
	• Trans-axial cines from neck to diaphragm	Detailed anatomy and connections
	• ***3D bSSFP with coronal orientation***	Detailed anatomy with high spatial resolution (around 1 mm)
**LV structure and Function**	• LV vertical long-axis cine, LVOT cine, AO valve cine, four chamber cine	
	• Short axis cine stack	Biventricular volumes and function
**RV structure and function**	• RV in out cine, RV LA cine, RVOT cine	RV function and signs of pulmonary hypertension
**PAs structure**	• RPA, LPA, Bifurcation cine	
**Flow Quantification**	• PC Through plane STJ aorta, MPA flow	Qp:Qs
	• PC Through plane RPA, LPA	Net flow differential
	• ***4D Flow***	Postprocessing includes flow assessments in the aorta (STJ), MPA, RPA and LPA, PVs
**Vascular anatomy**	• ***3D Contrast – Enhanced MR angiography***	Detailed vascular abnormalities (Pulmonary veins, Scimitar vein, APCs, Stenosis of scimitar vein pre- and post-operation, PAs distribution)
**Stress myocardial perfusion**	If clinically applicable – For example: Associated coronary abnormalities (anomalous of coronary origin pre- and post-operation, coronary fistula, APCs from coronary arteries)	Myocardial ischemia
**Late gadolinium enhancement**	If clinically applicable - For example: Associated coronary abnormalities (anomalous of coronary origin pre- and post-operation, coronary fistula, APCs from coronary arteries)	Myocardial infarction and scar

## 6 Limitations of each modality

Radiation exposure remains a key concern, especially in pediatric patients, where minimizing exposure is critical by utilizing many advanced techniques [[44], [45], [46]]. As such, CT is generally reserved for initial pre-operative and pre-catheter-intervention assessment and for specific follow-up questions when severe stenosis of a scimitar vein or reimplanted coronary artery or similar is suspected.

On the other hand, CMR can be challenging in smaller patients with high heart rates or those unable to hold their breath, as this may compromise image quality. The extended scanning time (approximately 40-60 min) required for full study can also be a limitation, particularly for patients with respiratory distress or severe heart failure. In some cases, general anesthesia may be necessary for small or uncooperative patients to ensure optimal imaging. Particularly small structures (severe stenosis of reimplanted veins etc.) can be beyond the resolution of CMR.

## 7 Optimizing imaging strategies

Due to the rarity of the condition, the wide spectrum of anatomical variants and clinical outcomes, there is no established consensus regarding intervention, treatment, or a standardized follow-up imaging strategy. The optimal pediatric cardiac imaging service comprises expert-led CMR and cardiac CT with close collaboration with clinicians in multidisciplinary meetings and attention to imaging protocols such that the right test is performed at the right time for the needs of each individual patient. This may include CT very early in life to avoid the need for general anesthesia and provide high detail pre-operative planning with the addition of CMR where extra information is required regarding split net flow or shunt calculation. For post-intervention follow-up or in cases managed conservatively, CMR may be performed at approximately 2-year intervals in stable, asymptomatic patients, or earlier if echocardiography suggests progression of right ventricular overload, with no additional radiation burden [47]. Cardiac CT is generally reserved for rapid imaging and targeted problem-solving in specific clinical scenarios, particularly in urgent situations. For example, it is useful in evaluating suspected stenosis or thrombosis of a scimitar vein baffle following surgery, to guide planning for potential rescue interventions.

## 8 Conclusion

In conclusion, SS is a rare congenital syndrome with a wide spectrum of variants. As such, management needs to be tailored for each individual. Advanced imaging plays a critical role in the comprehensive evaluation of SS in pediatric patients, offering detailed insights into both anatomy and hemodynamics, providing complementary information that is essential for accurate diagnosis, surgical planning, and long-term management. CT excels in fine detail anatomical assessment and rapid imaging in infants and young children, while CMR serves as the gold standard for non-invasive evaluation of hemodynamics and ventricular function and for shunt quantification. Multimodality imaging allows precise follow-up, ensuring that clinical decisions are based on comprehensive, longitudinal data for SS management.

## CRediT authorship contribution statement

**Thao V.N. Nguyen:** Writing – review & editing, Writing – original draft, Data curation, Conceptualization. **Hicran Gul Emral:** Writing – original draft. **Inga Voges:** Writing – review & editing, Validation, Funding acquisition. **S. Yen Ho:** Writing – review & editing, Validation. **Rigby L. Michael:** Writing – review & editing, Validation. **Piers E.J. Daubeney:** Writing – review & editing. **Edward Nicol:** Writing – review & editing. **Simon Padley:** Writing – review & editing. **Cemil Izgi:** Writing – review & editing. **Raad Mohiaddin:** Writing – review & editing, Validation. **Dudley J. Pennell:** Writing – review & editing. **Thomas Semple:** Writing – review & editing, Validation, Conceptualization. **Sylvia Krupickova:** Writing – review & editing, Validation, Supervision, Conceptualization.

## Declaration of competing interest

The authors declare the following financial interests/personal relationships which may be considered as potential competing interests:Dudley J. Pennell reports a relationship with Siemens that includes: funding grants. If there are other authors, they declare that they have no known competing financial interests or personal relationships that could have appeared to influence the work reported in this paper, other than SYH serving the IJCCHD Editorial Board, but had no involvement with the handling of this paper.
